# Determining an Optimal Cutoff of Serum β-Human Chorionic Gonadotropin for Assisting the Diagnosis of Intracranial Germinomas

**DOI:** 10.1371/journal.pone.0147023

**Published:** 2016-01-15

**Authors:** Hui Zhang, Peng Zhang, Jun Fan, Binghui Qiu, Jun Pan, Xi’an Zhang, Luxiong Fang, Songtao Qi

**Affiliations:** 1 Department of Neurosurgery, Nanfang Hospital, Southern Medical University, Guangzhou, 510515, China; 2 Department of Laboratory Medicine, Nanfang Hospital, Southern Medical University, Guangzhou, 510515, China; China Agricultural University, CHINA

## Abstract

**Background:**

Beta (β)-human chorionic gonadotropin (β-HCG) is used to confirm the diagnosis and plan treatment of intracranial germinomas. However, the cutoff values of serum β-HCG in diagnosis of intracranial germinomas reported in the literature are inconsistent. To establish an appropriate cutoff value of serum β-HCG for diagnosis of intracranial germinomas, we retrospectively reviewed the records of intracranial tumor patients who received serum β-HCG and α-fetoprotein (AFP) tests for diagnostic purposes at our hospital from 2005 to 2014.

**Methods:**

A total of 93 intracranial germinomas and 289 intracranial non-germ cell tumors were included in this study. Receiver operating characteristic (ROC) analysis was used to evaluate the sensitivity and specificity of 3 cutoffs (0.1, 0.4, and 0.5 mIU/mL) for diagnosing intracranial germinomas. The serum β-HCG level of intracranial germinoma patients was further analyzed to investigate the effect of metastasis status and tumor location on serum β-HCG level.

**Results:**

The area under the ROC curve was 0.81 (P < .001), suggesting β-HCG is an effective marker. Of the 3 cutoff values, 0.1 mIU/mL possessed a highest sensitivity (66.67%) and good specificity (91%). Although there was no β-HCG level difference between metastatic and non-metastatic intracranial germinoma patients, the diagnostic rate of metastatic neurohypophyseal germinomas was significantly higher than that of its non-metastatic counterpart (P < .05), implying that the location of the germinoma might need to be considered when β-HCG is used as a marker to predict metastasis.

**Conclusions:**

Determining an optimal cutoff of serum β-HCG is helpful for assisting the diagnosis of intracranial germinoma.

## Introduction

Intracranial germinomas are rare brain tumors with a geographically variable incidence, and account for 1–3% of all primary intracranial tumors in Western countries, but 4–10% in East Asia[1–3]. Intracranial germinomas mostly frequently develop in children and young adults[4].

According to the classification system of the World Health Organization (WHO), intracranial germinomas are a type of germ cell tumor (GCT). Other types of GCT are non-germinomatous germ cell tumors (NGGCTs) including embryonal carcinoma, choriocarcinoma, yolk-sac tumor, teratoma, and mixed tumors[5,6]. Intracranial germinomas are the most common type of GCT, accounting for up to 60% of all intracranial GCTs [7].

Intracranial germinomas commonly originate in the midline, including the pineal and neurohypophyseal regions [8].Other uncommon locations include the basal ganglia, the thalamus, the cerebral hemispheres, and the cerebellopontine angle[9]. There are different gender ratios depending on the location of the tumor. Pineal germinomas have a male to female ratio of 2.5:1, whereas neurohypophyseal germinomas develop more frequently in female than male patients [10]. Symptoms depend on the tumor location. Lesions in pineal region are associated with increased intracranial pressure and symptoms such as headache and vomiting [11], while neurohypophyseal germinomas usually cause diabetes insipidus and endocrine abnormalities [12].

Treatment options for intracranial germinomas include radiotherapy and chemotherapy. Compared to other intracranial tumors, intracranial germinomas are highly radiosensitive and potentially curable with radiotherapy alone [13–15]. Radiotherapy for intracranial germinomas results in a 10-year overall survival of more than 90% [16,17]. In addition, it has been shown that intracranial germinomas cannot be cured by surgical management [18]. Therefore, it is important to discriminate germinomas from other intracranial tumors at first diagnosis to develop an appropriate therapy plan. Due to lack of radioghraphic characteristics, diagnosis of intracranial germinoams mainly depends on the levels of tumor markers such as β-human chorionic gonadotropin (β-HCG) and α-fetoprotein (AFP) in serum and cerebrospinal fluid (CSF) [19,20]. In the case of a normal β-HCG level, pathogenic diagnosis is required [21].

β-HCG is produced by normal trophoblastic tissue in the placenta [22]. Serum levels are normally elevated in pregnant and postnatal women, and abnormally increased in patients with choriocarcinoma, and germ cell, bladder, pancreatic, gastric, and lung tumors [23]. One study has suggested that serum β-HCG have almost equal diagnostic value compared to CSF [24], however, serum is more readily available. Therefore, serum β-HCG is generally used as a first-line diagnostic marker and treatment planning.

However, the serum β-HCG cutoff value for the diagnosis of intracranial germinomas varies between studies. Institutions usually define their own normal range of serum β-HCG for intracranial germinomas. A cutoff value of β-HCG for diagnosis of intracranial germinomas has not been established, thus limiting its diagnostic value. Therefore, it is clinical significant to determine an appropriate cutoff value of β-HCG. Commonly reported cutoff values are 0.1 mIU/mL [25], 0.5 mIU/mL [26], 1 mIU/mL [27], 2.2 mIU/mL [28], 5 mIU/mL [29,30], and 6 mIU/mL [31]. Allen et al. [31] used a high cutoff of 6 mIU/mL, and concluded that there were only 5.5% germinomas patients with a β-HCG elevation.In another study, Lee et al. [29] used a cutoff of 5 mIU/mL and found only 30% germinomas patients with a β-HCG elevation.

To establish an appropriate cutoff value of serum β-HCG for diagnosis of intracranial germinomas, we retrospectively reviewed the records of intracranial tumor patients who received serum β-HCG and AFP tests for diagnostic purposes in our hospital during the recent 10 years. Based on confirmation of diagnosis, patients were grouped into an intracranial germinoma group and intracranial non-germ cell tumors group. Comparisons between the 2 groups were conducted to evaluate different cutoffs in term of the sensitivity and specificity for diagnosing intracranial germinomas. The serum β-HCG level in intracranial germinoma patients was further analyzed to investigate the effect of treatment, metastasis, and tumor location on serum β-HCG level.

## Materials and Methods

### Research design and patients

To evaluate the diagnostic value of serum β-HCG for intracranial germinomas, intracranial non-germ cell tumor patients were designed as a control group to compare with intracranial germinoma patients. We retrospectively reviewed the records of all intracranial tumor patients who received serum β-HCG and AFP testing for diagnostic purpose at Department of Neurosurgery, Nanfang Hospital, Southern Medical University from March 2005 to March 2014. Data extracted from the medical records included demographic information, pretreatment computed tomography (CT) and/or magnetic resonance imaging (MRI) images, pathological diagnosis, response to the radiotherapy/chemotherapy, follow-up data, and serum β-HCG and AFP levels. Patients were divided into the intracranial germinoma group (iGM group) and intracranial non-germ cell tumors group (INGCT group) according to the final diagnosis which was based on pathological and clinical data. This study was approved by the institutional review board of Nanfang Hospital, Southern Medical University.

### Inclusion criteria for iGM group

Criteria for inclusion in the iGM group were: 1) Pathologically confirmed diagnosis of intracranial germinoma; 2) Serum AFP level < 8.1 ng/mL (a common cutoff value for germinomas in the Chinese population [32]); 3) No intratumor hemorrhage or teratoma on pretreatment MRI and/or CT [33,34]; 4) Serum β-HCG level < 100mIU/mL; 5) Diagnostic radiotherapy with a dose of 20 Gy in the tumor area followed by MRI indicating the tumor size decreased by more than 50% [35]; and 6) In the case of residual tumor after radiotherapy, the residual tumor resolve during follow-up. Criteria for a pathological diagnosis were 1) and 2), while criteria for a clinical diagnosis were 2), 3), 4), 5), and 6).

### Exclusion criteria for iGM group

Exclusion criteria for the iGM group were: 1) Pathologically confirmed diagnosis of NGGCT; 2) Serum AFP level > 8.1 ng/mL; 3) Diagnosis of germinoma but with residual tumor after radiotherapy, and the tumor size did not continuously decrease followed by a pathological diagnosis of a NGGCT or not; 4) Diagnosis of germinoma without response to diagnostic radiotherapy and/or followed by a pathological diagnosis of non-germ cell tumor.

### Inclusion criteria for INGCT group

Inclusion criteria for the INGCT group were: 1) Postoperative pathological diagnosis of an INGCT; 2) Diagnosis of germinoma but no response to radiotherapy followed by postoperative pathological diagnosis of an INGCT; 3) Diagnosis of germinoma but no response to radiotherapy followed by a clinical diagnosis of an INGCT; 4) Diagnosis of a non-germinoma and with no response to radiotherapy followed by postoperative pathological diagnosis of an INGCT; 5) Diagnosis of a non-germinoma and no response to radiotherapy followed by a clinical diagnosis of an INGCT.

### Exclusion criteria for INGCT group

The cases excluded from this study are shown below and were: 1) Metastatic breast, lung, and ovarian tumors to the brain; 2) Postnatal meningioma; 3) Postnatal cavernous hemangioma; 4) Metastatic gestational choriocarcinoma to the brain.

### Serum β-HCG level determination

Blood samples were collected into a clot activator tube, and after clotting the samples were centrifuged at 2,000 *g* for 10 minutes. The resulting supernatant was collected, and serum β-HCG level was determined by a Cobas E601 Immunology Analyzer (Roche, Switzerland) and a β-HCG assay kit according to the manufacture’s protocol. This assay has a good within-batch imprecision (< 2.8%), between-batch imprecision (< 7.4%), and functional sensitivity (< 0.1 mIU/mL), and a linear range of 0.1~10000 mIU/mL.

### Statistical Analysis

Age was presented as median (minimum, maximum). Other continuous data were presented as mean ± standard deviation (SD), while categorical data were displayed as number and percentage (%). Due to the measurement limitation of β-HCG, all data which were recorded as < 0.1 were transformed to 0.099 for analysis. Receiver operating characteristic (ROC) curve analysis and corresponding statistics were utilized for predicting iGM classification based on serum β-HCG level. Student’s t-test (independent and paired), Mann-Whitney U test, Wilcoxon signed-rank test, phi coefficient, and Chi-square test were used depending on the properties of the data. All analyses were performed using IBM SPSS Version 20 (SPSS Statistics V20, IBM Corporation, Somers, New York). A value of *P* < .05 was considered to indicate statistical significance.

## Results

### Patient demographic data

A total of 382 patients were included in the analysis: 93 in the iGM group and 289 in the INGCT group. The iGM group had a median age of 16 years (range, 2–58 years), while the INGCT group had a median age of 21 years (rang, 0–79 years), and the age difference between the 2 groups was statistical significant (*P* < .01). The percentage of males in the iGM and INGCT group was 80.6% and 55.7%, respectively, and the difference was significant (*P* < .01).

### β-HCG is an effective tumor marker for intracranial germinomas

**Fig 1:**
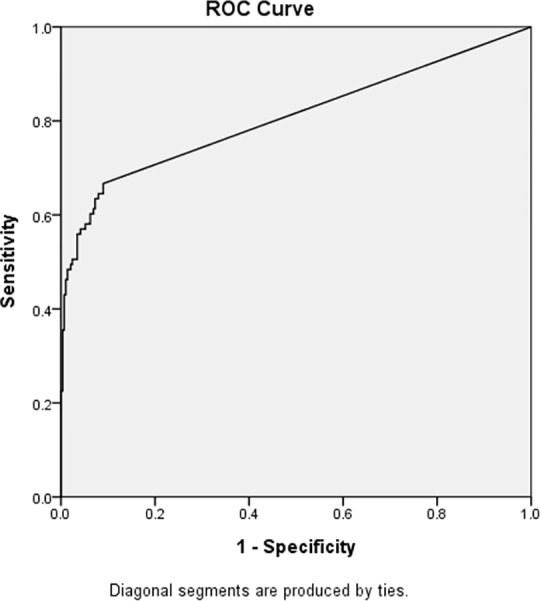
Diagnosis of intracranial germinoma by pre-treatment serum β-human chorionic gonadotropin (β-HCG) level. Area under the receiver operating characteristic curve = 0.81 (*P* < 0.001).

The ROC curve for β-HCG as a tumor marker for diagnosis of intracranial germinomas is shown in Fig 1. The area under the ROC curve (AUC) was 0.81 (*P* < .001, standard error = 0.03, 95% confidence interval [CI]: 0.75–0.87), suggesting that serum β-HCG is an effective tumor marker for diagnosis of intracranial germinomas.

### Optimal cutoff value of serum β-HCG for intracranial germinoma diagnosis

Since the ROC analysis revealed that serum β-HCG is an effective tumor marker for diagnosis of intracranial germinomas, we attempted to determine the appropriate cutoff value for serum β-HCG. We analyzed 2 commonly used cutoffs for serum β-HCG, 0.1 and 0.5 mIU/mL, and an intermediate cutoff value of 0.4 mIU/mL. The diagnostic rate (the percentage of patients with a β-HCG level > cutoff), phi coefficient, and diagnostic statistics of the ROC analysis for the 3 cutoff values are shown in Table 1. The difference in diagnostic rate between the 2 groups for all cutoff values was statistical significant (*P* < .01), and had a positive phi coefficient. All 3 cutoff values exhibited good specificity (> 90%) and a moderate sensitivity (55.91–66.67%). These data demonstrate that the 3 cutoff values are all appropriate for diagnosis of intracranial germinomas. Among them, the value of 0.1 mIU/mL had a highest sensitivity (66.67%), therefore we chose it as the cutoff value for subsequent analyses.

**Table 1:** Diagnostic rate and ROC statistics of different β-HCG cutoffs.

Cutoff	0.1 mIU/mL	0.4 mIU/mL	0.5 mIU/mL
	**Diagnostic rate**^a^		
iGM	62 (66.67%)*	54 (58.06%)*	52 (55.91%)*
INGCT	26 (9.00%)	16 (5.54%)	11 (3.81%)
ψ	0.59	0.58	0.6
	**ROC statistics**		
Sensitivity	66.67%	58.06%	55.91%
Specificity	91.00%	94.46%	96.19%
PLR	7.41	10.49	14.69
NLR	0.37	0.44	0.46
PPV	70.45%	77.14%	82.54%
NPV	89.46%	87.50%	87.15%

^a^ The percentage of β-HCG levels higher than the cutoff value.

^*^P < .01 compared with INGCT group.

Abbreviation: iGM, intracranial germinoma; INGCT, intracranial non-germ cell tumors; PLR, positive likelihood ratio; NLR, negative likelihood ratio; PPV, positive predictive value; NPV, negative predictive value; ROC, receiver operating characteristic; β-HCG; β-human chorionic gonadotropin.

### Change of serum β-HCG after treatment of intracranial germinoma

Next, we examined if the serum β-HCG level would change after treatment of intracranial germinomas. Within the iGM group, serum β-HCG levels pre- and post-treatment (radiotherapy with or without chemotherapy) were available in 40 paitents. Of them, 37 patients had a pre-treatment serum β-HCG ≥ 0.1 mIU/mL, and a post-treatment level < 0.1 mIU/mL. The other 3 patients entered our center with the diagnosis of recurrent germinomas based on radiographic data and serum β-HCG levels of 0.11 2.2, and 11.2 mIU/mL, respectively. Their β-HCG levels after retreatment were 0.19, 0.57, and 7.2 mIU/mL, respectively, and the radiographic data after retreatment comfirmed failure of treatment. These results further indicate that 0.1 mIU/mL is an appropriate cutoff for the diagnosis of intracranial germinomas.

### Differences between metastatic and non-metastatic intracranial germinoma patients

The relationship between metastasis and serum β-HCG level in intracranial germinoma patients is still unclear. To investigate if there was any difference between serum β-HCG levels of metastatic and non-metastatic germinomas, the 93 germinoma patients were grouped according to the presence of metastatic disease. As shown in Table 2, there was no significant difference in the pre-treatment β-HCG level between the 2 groups (*P* > .05). However, the difference in age was significant between individual with metastasis and those without metastasis (*P* < .01).

**Table 2:** Comparison between metastatic and non-metastatic patients in the iGM group.

	N	Pre-treatment β-HCG (mIU/mL) mean±SD	Age (years) Median (range)
Non-metastasis	68	32.62±95.5	15 (2, 39)^*^
Metastasis	25	25.62±81.83	18 (2, 58)

^*^P < .01 compared with metastatic group.

iGM, intracranial germinoma; β-HCG; β-human chorionic gonadotropin; SD, standard deviation.

We further investigated if the location of the intracranial germinoma has an effect on the relationship between metastasis and the β-HCG level. The 93 intracranial germinoma patients were grouped according to tumor location and metastatic status (Table 3). The difference in diagnostic rate between non-metastatic pineal germinomas and metastatic pineal germinomas was not significant (69% vs. 59%, *P* = .45), while the difference in diagnostic rate between non-metastatic bifocal intracranial germinomas and metastatic bifocal intracranial germinomas was significant (29% vs. 100%, *P* = .028). The diagnostic rates of non-metastatic neurohypophyseal germinomas and metastatic neurohypophyseal germinomas were 63% and 100%, respectively, but the difference was not statistical significant (*P* = .533), most likely due to the small sample size of metastatic neurohypophyseal germinomas (n = 2).We therefore combined neurohypophyseal and bifocal germinomas (simultaneous involvement of the pineal and the neurohypophyseal regions) to analyze the difference of diagnostic rates between metastatic and non-metastatic disease, and the difference between these 2 group was significant (53.8% vs. 100%, *P* = .027). These results imply that tumor location might need to be considered when investigating the relationship between the metastatic status and pre-treatment serum β-HCG level in intracranial germinomas. On the other hand, the chi-square test showed a significant gender difference among all groups (*P* < .01).

**Table 3:** β-HCG diagnostic rate of intracranial germinomas by location and metastatic status.

Tumor	Diagnostic rate (cutoff of 0.1 mIU/mL)	N	Male**	Female
**Metastatic cerebellar vermis germinoma**	100%	1	0	1
**Non-metastatic basal ganglia germinomas**	100%	3	3	0
**Non-metastatic neurohypophyseal germinomas**	63%	19	9	10
**Metastatic neurohypophyseal germinomas**	100%	2	2	0
**Non-metastatic pineal germinomas**	69%	39	15	2
**Metastatic pineal germinomas**	59%	17	36	3
**Non-metastatic bifocal intracranial germinomas**	29%	7	6	1
**Metastatic bifocal intracranial germinomas**	100%^*^	5	4	1
**Total**	66%	93	75	18

^*^P < .05 compared with non-metastatic counterpart.

^**^P < .01, gender difference among all groups.

## Discussion

Typically, the comprehensive diagnostic criteria for suspected germinomas in our center include age, sex, the origin of tumor identified by MRI, exclusion from the possibility of teratoma and choriocarcinoma by MRI, β-HCG and AFP level. According to the comprehensive diagnosis, the patients with suspected intracranial non-germ cell tumors will receive tumor resection and pathologically confirmed diagnosis. For patients with suspected NGGCTs or mixed germ cell tumors, individual treatment will be carried out. The patients with suspected germinomas will receive diagnostic radiotherapy and then the treatment strategy may be modified according to the therapeutic outcome of the radiotherapy. The tumors characteristics will be confirmed finally during the follow-up. In addition, in the western countries, germinomas require neurosurgical biopsy to obtain a histological diagnosis [36]. The suspected germinoma cases were comprehensive diagnosed by serum β-HCG level and other above clinical data, and then underwent surgery for biopsy other than tumor resection. There are no other tumor markers better than β-HCG and AFP for now, therefore determining an optimal cutoff of serum β-human chorionic gonadotropin for assisting the diagnosis of intracranial germinomas is clinical significant.

To the best of our knowledge, this is the first study aiming to determine an appropriate cutoff value of serum β-HCG for the diagnosis of intracranial germinomas with a large sample size. A total of 93 intracranial germinoma and 289 intracranial non-germ cell tumors patients were included to determine an optimal cutoff value of serum β-HCG for intracranial germinomas. There were age and gender differences between the iGM and INGCT groups, and these differences can be attributed to the age and gender preferences of intracranial germinomas. It has been shown that males have significantly higher rates of intracranial GCTs than females, and intracranial germinomas peaked in incidence during adolescence [37].

Our study showed that a cutoff of 0.1 mIU/mL had a sensitivity of 66.67%, while a cutoff of 0.5 mIU/mL had a sensitivity of 56.2%, suggesting a lower cutoff value would lead to a higher sensitivity. However, the sensitivity cannot be elevated to a satisfactory value without limit since some intracranial germinomas secret no or extremely low levels of β-HCG. The germinoma category includes germinomas with syncytiotrophoblast giant cells, which secrete β-HCG, and pure germinomas that do not secrete β-HCG [38]. The non-secreting pure germinomas are the main reason for the low sensitivity of β-HCG. However, even with limitations in sensitivity, serum β-HCG is still useful for the diagnosis. A lower cutoff can increase the sensitivity, but also decrease the specificity (increase the number of false positive diagnosis). In our study, the 3 cutoffs examined all had a high specificity (91–96.19%).

For a cutoff of 0.1 mIU/mL there were 26 false positive patients in the INGCT group. Further analysis found that there were significant age (Mann-Whitney Test, *P* < .01) and gender (chi-square test, *P* < .01) differences between INGCT patients with a β-HCG value ≥ 0.1 mIU/mL (false positive group) and with a β-HCG value of < 0.1 mIU/mL (negative group). The gender ratios (males to females) and median age were 0.37 vs. 1.41, and 49 years (range, 2–79 years) vs. 20 years (range, 0–75 years) for the false positive and negative group, respectively. These results imply that female and elderly patients might have a higher chance of having a false positive β-HCG result. It is worth noting that, there was only 1 of 26 false positive patients in the INGCT group originated in the pineal gland, which is a 56 years old female with pinealocytom.For a cutoff of 0.5 mIU/mL, there were 11 false positive cases in the INGCT group, which included a 17 years old female, a 66 years old male, and 9 females aged 44–65.

It is worth noting that in this study 73.1% of germinomas originated inthe pineal gland. Compared lesions in the neurohypophyseal region, those in the pineal region have a significantly higher male/female ratio [39]. Germinomas peak in incidence during adolescence [37]. Based on the above observations, the cutoff of 0.1 mIU/mL may be more reliable for diagnosis of pineal germinomas in male adolescent patients. However, before the findings of this study being verified by studies with a large sample size, we recommend that the cutoff value of 0.5 mIU/mL could be used in assisting the diagnosis of germinomas since it had a highest specificity (96.16%).

We found that the β-HCG level declined significantly after treatment. Of the 40 cases examined, 37 had a β-HCG ≥ 0.1 mIU/mL before treatment and the level decreased to < 0.1 post treatment. This result is consistent that of prior study [40]. A change in β-HCG level is more sensitive imaging changes, and is used as an indicator of treatment efficacy during follow-up [41]. In the case of a β-HCG level < 0.1 mIU/mL after treatment, and > 0.1 during follow-up recurrence should be considered. After radiotherapy, if the serumβ-HCG level is still higher than 0.1 mIU/mL and the case is excluded from a false positive one, a new tumor treatment strategy is required.

Our data demonstrated no difference in β-HCG level between metastatic and non-metastatic patients. Although a high β-HCG was previously believed to be a marker of poor prognosis of intracranial germinomas [42], recent study has suggested this is not the case [43]. A number of studies have suggested that β-HCG level cannot be used to predict the recurrence rate [44,45], or prognosis [46,47] of intracranial germinomas. We found that β-HCG was not correlated with metastasis of intracranial germinomas, which is consistent with recent literature. Our data also showed that the non-metastatic patients had a lower median age than metastatic patients. However, it remains to be determined if this difference has clinical significance.

Although there was no significance difference in β-HCG level between metastatic and non-metastatic intracranial germinomas, we found a significant difference in the diagnostic rate of β-HCG among different locations and metastatic status of intracranial germinomas. Analysis found the diagnostic rate of β-HCG for metastatic neurohypophyseal germinomas (including bifocal germinomas) was significantly higher than the rate for the non-metastatic counterpart (100% vs.54%, *P* < .05). A similar difference was not observed in pineal germinomas. This observation suggests that if an elevated serum β-HCG is found in patients with neurohypophyseal germinomas, metastasis should be considered. However, it remains be to determined whether this difference is due to characteristics of germinomas per se, or sampling errors.

In summary, we evaluated the diagnostic value of serum β-HCG for intracranial germinomas in 93 intracranial germinomas patients and 289 control patients. ROC curve analysis demonstrated that β-HCG is an effective tumor marker for intracranial germinomas. A cutoff of 0.1 mIU/mL possessed a good sensitivity (66.67%) and a moderate specificity (91%). A total of 92.5% (37/40) of patients had a pre-treatment serum β-HCG > 0.1 mIU/mL, and a post-treatment level < 0.1 mIU/mL. Taken together, 0.1 mIU/mL is an optimal cutoff of serum β-HCG for the diagnosis of intracranial germinomas. Lastly, it is warranted to conduct further ROC analyses with a larger sample size to determine a cut-off value of serum β-HCG for the diagnosis of iGM with different pathological stages.
